# Gut Microbiome Biomarkers and Functional Diversity Within an Amazonian Semi-Nomadic Hunter–Gatherer Group

**DOI:** 10.3389/fmicb.2019.01743

**Published:** 2019-07-30

**Authors:** Liliane Costa Conteville, Joseli Oliveira-Ferreira, Ana Carolina Paulo Vicente

**Affiliations:** ^1^Laboratory of Molecular Genetics of Microorganisms, Oswaldo Cruz Institute, Oswaldo Cruz Foundation, Rio de Janeiro, Brazil; ^2^Laboratory of Immunoparasitology, Oswaldo Cruz Institute, Oswaldo Cruz Foundation, Rio de Janeiro, Brazil

**Keywords:** shotgun metagenomic sequencing, human gut microbiome, hunter–gatherers, Yanomami, Amerindian, taxonomic biomarkers, gut functionality, gut microbes

## Abstract

Human groups that still maintain traditional modes of subsistence (hunter–gatherers and rural agriculturalists) represent human groups non-impacted by urban-industrialized lifestyles, and therefore their gut microbiome provides the basis for understanding the human microbiome evolution and its association with human health and disease. The Yanomami is the largest semi-nomadic hunter–gatherer group of the Americas, exploring different niches of the Amazon rainforest in Brazil and Venezuela. Here, based on shotgun metagenomic data, we characterized the gut microbiome of the Yanomami from Brazil and compared taxonomically and functionally with the Yanomami from Venezuela, with other traditional groups from the Amazon and an urban-industrialized group. Taxonomic biomarkers were identified to each South American traditional group studied, including each Yanomami group. Broader levels of functional categories poorly discriminated the traditional and urban-industrialized groups, but the stratification of these categories revealed clear segregation of these groups. The Yanomami/Brazil gut microbiome presented unique functional features, such as a higher abundance of gene families involved in regulation/cell signaling, motility/chemotaxis, and virulence, contrasting with the gut microbiomes from the Yanomami/Venezuela and the other groups. Our study revealed biomarkers, and taxonomic and functional features that distinguished the gut microbiome of Yanomami/Brazil and Yanomami/Venezuela individuals, despite their shared lifestyle, culture, and genetic background. These differences may be a reflection of the environmental and seasonal diversity of the niches they explore. Overall, their microbiome profiles are shared with South American and African traditional groups, probably due to their lifestyle. The unique features identified within the Yanomami highlight the bias imposed by underrepresented sampling, and factors such as variations over space and time (seasonality) that impact, mainly, the hunter–gatherers.

## Introduction

The transition from the traditional modes of subsistence (hunter–gatherers/rural agriculturalists) to the current western lifestyles that occurred with the advent of modern practices (urbanization and industrialization) brought wide differences in diet and environment; factors proposed to be determinants of the gut microbiome composition (Gupta et al., 2017). In fact, cross-population studies have demonstrated distinct taxonomic and functional profiles between the gut microbiome of hunter–gatherers/rural agriculturalists and urban-industrialized human groups. The main differences between the gut microbiome of these groups are that hunter–gatherers/rural agriculturalists individuals harbor a more diverse gut microbiome, with higher levels of fiber-degrading bacteria, and unique taxa that are depleted in the urban-industrialized populations (De Filippo et al., 2010, 2017; Yatsunenko et al., 2012; Schnorr et al., 2014; Clemente et al., 2015; Martínez et al., 2015; Obregon-Tito et al., 2015; Rampelli et al., 2015; Gomez et al., 2016; de la Cuesta-Zuluaga et al., 2018; Li et al., 2018; Rothschild et al., 2018). Studies focusing on the gut microbiome differences of traditional and urban-industrialized groups reveal under-explored scenarios that can expand the field of prebiotics and probiotics for modern disorders prevention and treatment, as well as biomarkers that can form the basis for health and prognostic disease tests (Schnorr et al., 2014; de la Cuesta-Zuluaga et al., 2018). Considering that human groups that live in a non-western lifestyle are in decline, the study of the remaining traditional groups constitutes an extraordinary opportunity to explore and unravel the human gut microbiome before modernization.

**FIGURE 1:**
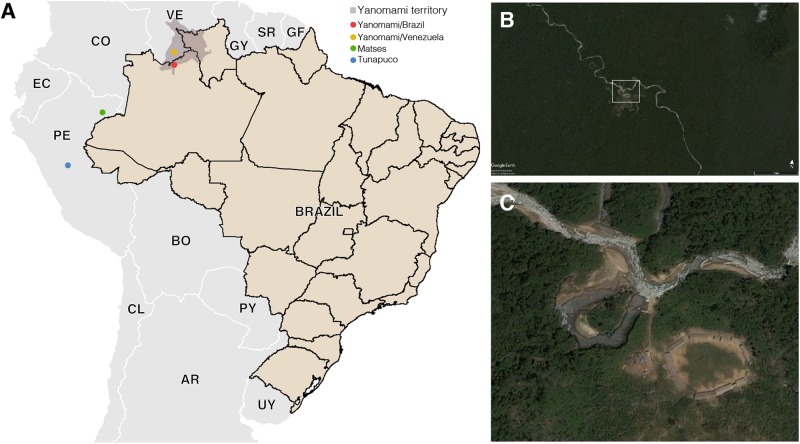
**(A)** Geographic locations of the South American traditional groups. **(B,C)** Satellite image of a Yanomami village in the Brazilian Amazon. *Source*: Google Earth and Instituto Socioambiental (https://acervo.socioambiental.org/).

The Yanomami is the largest indigenous semi-isolated group in the Amazon to maintain traditional subsistence practices based on hunting, fishing, gathering, and swidden horticulture (Albert and Le Tourneau, 2007). They inhabit the Amazon region encompassing the Brazil and Venezuela border, living in villages located at sea level as well as on high mountains in a huge area in the Amazon (Pithan et al., 1991). Their diet is low in fat and salt, and high in fruits, fiber, and sylvatic animals. Common chronic diseases in modern societies, as atherosclerosis and obesity are virtually unknown among Yanomami (Mueller et al., 2018). In order to go deeper and enlarge the characterization of hunter–gatherers gut microbiome, we generated shotgun metagenomic data of semi-isolated Yanomami from Brazil, and performed comparative analyses with data from the remote Yanomami from Venezuela (Clemente et al., 2015), other traditional groups from the Amazon, the Matses, and the Tunapuco (Obregon-Tito et al., 2015), as well as an urban-industrialized group (Human Microbiome Project Consortium, 2012; Lloyd-Price et al., 2017; Figure 1). These traditional groups live and explore different niches of the Amazon Region, which includes the most extensive and preserved rainforest in the world (the Amazon Rainforest), vast areas of scrub-savannah, as well as the Andes highlands (Sioli, 1984; Verìssimo and Capobianco, 2001). This variable geomorphology, climate, and vegetation cover result in a diverse diet and environment for these traditional groups.

We hypothesized that the diversity in the ecological niches and diet would impact the gut microbiome composition and functionality, expressing the adaptation to evolutionary and environmental constraints of each site inhabited, despite being traditional groups and sharing the cultural and genetic background. Our results confirmed our hypothesis, demonstrating that even within Yanomami, there was a diversity of taxonomic biomarkers and unique functional features.

## Materials and Methods

### Study Participants and Sample Collection

The protocol of this study was reviewed and approved by the Oswaldo Cruz Foundation’s Ethics Research Committee No. 638/11 and by the National Ethics the Committee in Research – CONEP No. 16907. Before participating in the study, a bilingual interpreter (a Yanomami native who spoke Portuguese) explained to the leaders and/or indigenous representatives, the purpose and importance of the study, the procedures to be carried out, and finally requested permission by fingerprint consent of each participant. Participants were requested to provide a morning fecal sample, and a labeled screw-capped plastic container was provided. A single stool sample was collected from each subject on the following day, and samples were stored in separate sterile feces containers. At the time of the collection, age and gender information of the individuals were also acquired. These details are summarized in Supplementary Table 1.

### DNA Extraction, Library Preparation, and Sequencing

Total DNA was extracted from 15 stool samples with FastDNA^®^ SPIN Kit (MP Biomedicals), following the manufacturer’s instructions. The DNA concentration was evaluated using Qubit^®^ 2.0 Fluorometer (Life Technologies). Metagenomic libraries were constructed with TruSeq DNA Sample Preparation v2 Kit following the standard protocols. Purified libraries were sequenced on a HiSeq^®^ 2500 sequencer (Oswaldo Cruz Foundation, high-throughput sequencing platform) in two batches, producing a total of ∼219 million reads, with an average of ∼14 million reads per sample.

### Bioinformatics Processing

Raw reads were trimmed and filtered (phred quality < 20, length < 30) using Trimmomatic (Bolger et al., 2014). The remaining reads (∼206 million reads) were mapped to a human reference genome (Hg38) using Bowtie2 (Langmead and Salzberg, 2012). Non-host reads (∼198 million reads) were used in further analysis. Besides the metagenomes generated in this study, we also analyzed shotgun metagenomic data from previously published studies: two hunter–gatherer communities [Yanomami from Venezuela, *n* = 8 (Clemente et al., 2015); Matses, *n* = 24 (Obregon-Tito et al., 2015)], a rural agricultural community [Tunapuco, *n* = 12 (Obregon-Tito et al., 2015)], and urban populations [United States, *n* = 44 (Human Microbiome Project Consortium, 2012; Lloyd-Price et al., 2017)]. These datasets were sequenced on Illumina platforms, and bioinformatic processing was performed in parallel with the data generated in this study.

The taxonomic classification was performed by Kraken (Wood and Salzberg, 2014), using a database of whole genomes of bacteria and archaea from NCBI. Linear discriminant analysis (LDA) was performed using LEfSe (Segata et al., 2012) on genus-level relative abundance table to detect bacterial genera that characterize the differences between the groups. For LEfSe analysis, the Kruskal–Wallis test (alpha value of 0.05) and LDA score of >4.0 were used as thresholds. Functional classification was classified by SUPER-FOCUS (Silva et al., 2016) based on the genes families from the SEED database.

### Statistical Analysis

For general data manipulation and statistical analysis, we employed the vegan (Oksanen et al., 2013) and phyloseq (McMurdie and Holmes, 2013) packages in R. Shannon index of alpha-diversity was estimated for each metagenome, with pairwise Wilcoxon test being used for statistical difference evaluation. Beta-diversity was determined using Bray–Curtis dissimilarity and permutational multivariate analysis of variance (PERMANOVA) was performed with 999 permutations to estimate a *P*-value for differences among traditional and westernized groups.

## Results

### Lifestyle of the Human Group Studied

The Yanomami group is mainly hunter–gatherers, but in some communities, the women cultivate plantains and cassava, while men go hunting. Their diet consists on the variety of foods seasonally available in the rainforest, which includes all kinds of edible fare ranging from snakes, wild pigs, monkeys, deer, and jaguars to insects, larvae, fish, crabs, wild honey, roots, and palm fruits. They live in large communal huts constructed mainly of thatched palm leaves and wood, which are shared by the entire village. The drinking water is collected directly from unprotected wells and river streams (personal communication).

### Intra- and Inter-Individual Diversity of the Gut Microbiomes

To unravel the gut microbiome diversity of Yanomami from Brazil individuals (Yanomami/Brazil, *n* = 15), we performed alpha- and beta-diversity analyses based on the bacterial genera profile identified by Kraken. Association between gut microbiome composition and gender was analyzed with principal coordinate analysis (PcoA) generated with Bray–Curtis distances. These comparisons did not reveal any statistically significant differences in the gut microbiome of female and male Yanomami/Brazil individuals (PERMANOVA, *P* > 0.5, Supplementary Figure 1). To compare the alpha- and beta-diversity of the Yanomami/Brazil with other groups, we also reanalyzed and compared gut microbiome data gathered from other South American traditional communities: the Yanomami from the Venezuelan Amazon (Yanomami/Venezuela, *n* = 8) (Clemente et al., 2015), the Matses from the Peruvian Amazon (*n* = 24) (Obregon-Tito et al., 2015), the Tunapuco from the Andean highlands (*n* = 12) (Obregon-Tito et al., 2015); and a representative group of urban individuals from the United States (*n* = 44) (Human Microbiome Project Consortium, 2012; Lloyd-Price et al., 2017).

**FIGURE 2:**
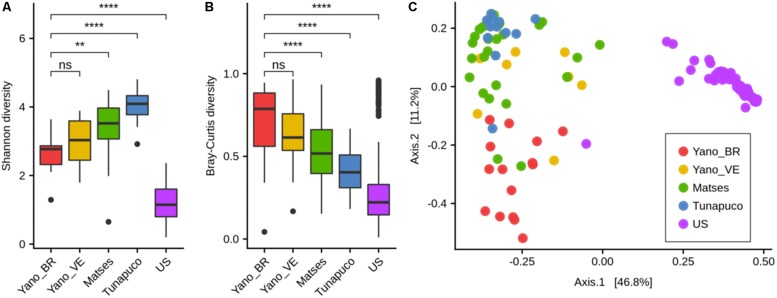
Alpha- and beta-diversity comparisons of the gut microbiomes of each group. Analyses were performed on genus-level taxa tables. ns, not significant; ^∗∗^*P* < 0.01, ^****^P < 0.0001 (Wilcoxon test). **(A)** Boxplot of the Shannon diversity of each group. **(B)** Bray–Curtis distances within each group. **(C)** Principal coordinate analysis of Bray–Curtis distances. The colors of the boxplots and dots represent the different groups analyzed according to the legend. Yano_BR, Yanomami/Brazil; Yano_VE, Yanomami/Venezuela; US, US individuals.

There was no statistical difference between the Yanomami/Brazil and Yanomami/Venezuela regarding intra- and inter-diversity (alpha- and beta-diversity, respectively) of the gut microbiome. The Yanomami individuals showed the lowest bacterial alpha-diversity among the traditional groups, and all the traditional human groups presented higher bacterial diversity compared to the urban individuals (Figure 2A). Regarding the beta-diversity, the Yanomami individuals presented the highest interpersonal variation, and the urbans presented the lowest (Figure 2B). Clear segregation was observed among the semi-isolated and westernized individuals (PERMANOVA, *P* = 0.001) based on PcoA generated with Bray–Curtis distances. In addition, higher dispersion of Yanomami/Brazil and Yanomami/Venezuela samples was observed, stressing their higher interpersonal variation (Figure 2C).

### Microbiomes Taxonomic Characterization

**FIGURE 3:**
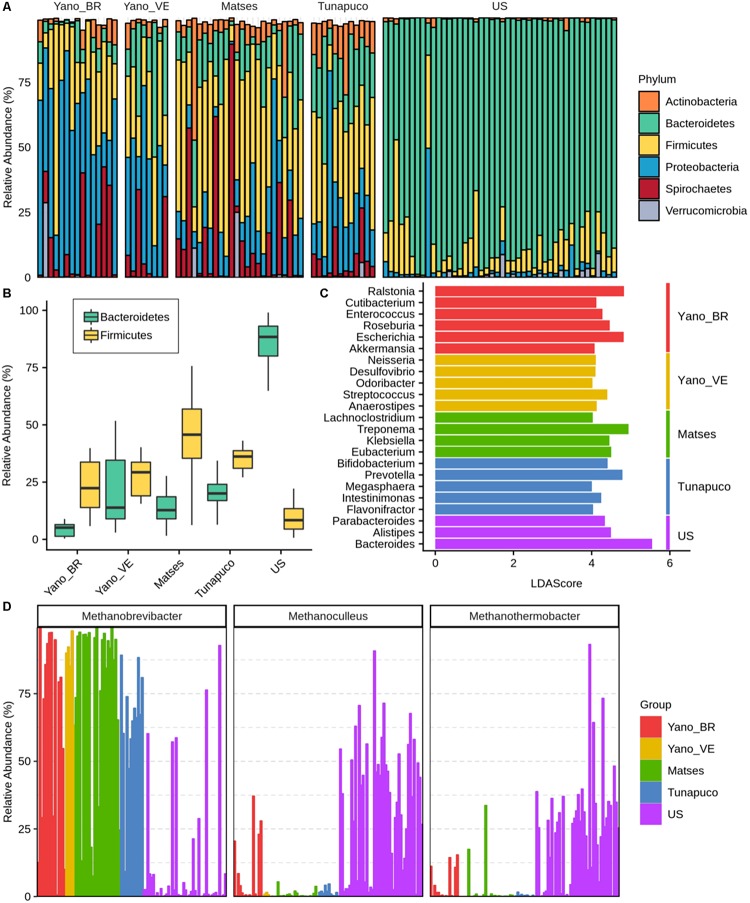
Bacterial and archaeal taxa differences among traditional and urban groups. **(A)** Barplot representing the relative abundance (percentage) of the most frequent phyla in the gut microbiomes. **(B)** Boxplots showing the Bacteroidetes and Firmicutes abundance (percentage) in each group. **(C)** Bar chart showing the LDA scores >4 of bacterial genera found to be significantly associated with each group. **(D)** Relative abundance of the most prevalent archaeas identified in the groups. Yano_BR, Yanomami/Brazil; Yano_VE, Yanomami/Venezuela; US, US individuals.

In order to identify which bacterial and archaeal taxa differentiate the traditional groups from the urban group, the microbiomes were compared at both phylum and genus scales. Thirty-two bacterial phyla were identified, with 16 phyla having significant differences in the relative abundances among the groups (Kruskal–Wallis test: *P* < 0.0001). The relative abundance of the bacterial genera identified in each Yanomami/Brazil gut microbiome is shown in Supplementary Table 2. Considering the traditional and urban groups, a clear difference at phylum level was observed, with the former having higher biodiversity characterized by Firmicutes, Proteobacteria, Bacteroidetes, and Spirochaetes, and the urban group being mainly characterized by Bacteroidetes (Figure 3A).

The Yanomami/Brazil as well as the other traditional individuals follow a trend in which they have higher Firmicutes and lower Bacteroidetes levels, while the opposite was observed in the urban individuals (Figure 3B). The Firmicutes in each traditional group was characterized by distinct genera, with no prevalent genus consistently observed in the groups. In fact, all traditional groups presented different genera from the Firmicutes phylum as biomarkers (Figure 3C). Genera from Bacteroidetes phylum were demonstrated to be the biomarkers of the urban group (Figure 3C).

Distinctly from the other groups, Proteobacteria was the most prevalent phylum among the Yanomami individuals, despite their geographic origin (Brazil and Venezuela). The most abundant genera of this phylum in the traditional groups were *Escherichia* and *Klebsiella*; however, there is a contrasting higher abundance of *Escherichia* and *Ralstonia* genera in the Yanomami/Brazil, and therefore, they were defined as Yanomami/Brazil biomarkers. On the other hand, *Neisseria* and *Desulfovibrio* were defined as Yanomami/Venezuela biomarkers, while *Klebsiella* was the biomarker of the Matses group. It is noteworthy that *Cutibacterium* from the Actinobacteria phylum and *Akkermansia* from the Verrucomicrobia phylum were also deemed as the biomarkers of the Yanomami/Brazil (Figure 3C). Besides that, the Yanomami/Brazil, similarly with the other semi-isolated, present *Treponema* and *Brachyspira*, two genera from the Spirochaetes that were not detected in the urban group.

Concerning Archaea, we observed that the most abundant genus in the traditional groups was *Methanobrevibacter*, comprising ∼70% of all archaea classified reads, while in the urban population, there was a high abundance of *Methanoculleus* and *Methanothermobacter*, all archaea methane-producers (Figure 3D).

### Microbiomes Functional Characterization

**FIGURE 4:**
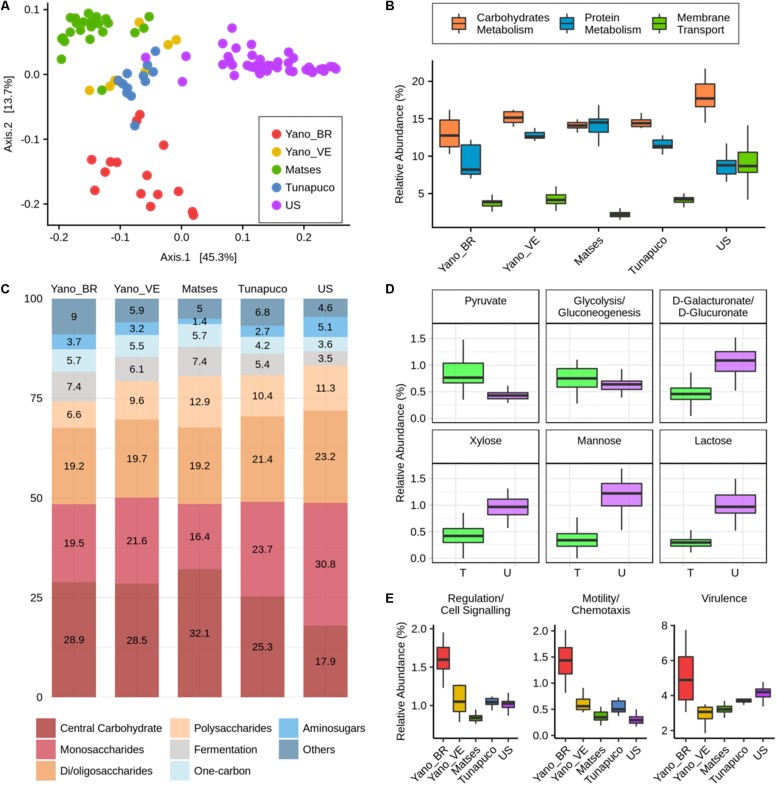
Functional metabolic characteristics of the traditional and urbanized microbiomes. **(A)** Principal coordinate analysis of Bray–Curtis distances based on functions at the level 3 subsystems. **(B)** Boxplots showing the abundance (percentage) of the level 1 main functions of each group. **(C)** Barplot representing and showing the mean relative abundance (percentage) of the main level 2 functions within carbohydrates metabolism in each group. **(D)** Boxplots showing the abundance (percentage) of level 3 functions, considering the traditional groups as one group, “T,” compared to the urban group, “U.” **(E)** Boxplots showing the abundance (percentage) of the level 1 functions that are enriched in the Yanomami/Brazil. Yano_BR, Yanomami/Brazil; Yano_VE, Yanomami/Venezuela; US, US individuals.

For functional characterization, the metagenomic reads of all groups were assigned to gene families from the SEED database and were categorized for their functional roles in subsystems with three levels of resolution, in which level 1 represents the broader category. We observed segregation between the traditional and urban groups regarding the abundance of functions at level 3. Interestingly, among the traditional groups, the Matses and Yanomami/Brazil individuals exhibit clear segregation; however, the Yanomami/Brazil present a more disperse pattern, indicating a quite diverse functional characteristic concerning functions at level 3 (Figure 4A).

The most abundant metabolic functions present in the microbiome of all groups at level 1 were the metabolism of carbohydrates and proteins, and in addition, membrane transport was also a main function in the urban group, which was significantly reduced in the traditional groups (*P* < 2*e*−05, Wilcoxon rank-sum test, Figure 4B). At level 2, differences within the carbohydrate metabolic functions occur between the traditional and the urban groups, which the main functions in the traditional groups belonged to the central carbohydrate metabolism, while in the urbans, the major functions were the monosaccharides and di-/oligosaccharides metabolism (Figure 4C). There was no difference among the groups’ microbiome within the protein metabolism, in which the most abundant functions are those associated with biosynthesis and degradation of proteins (Supplementary Figure 2).

At level 3, comparing the group of traditionalists and urbans, it was possible to observe a significantly enrichment within the central carbohydrate metabolism functions: pyruvate metabolism and glycolysis/gluconeogenesis, in the former (*P* = 1.3*e*−13 and *P* = 0.0031, respectively, Wilcoxon rank-sum test, Figure 4D). In monosaccharides metabolism, there were also differences between the groups: D-galacturonate/D-glucuronate and xylose utilization were the most prevalent functions in the traditional groups, while mannose metabolism was the most prevalent in the urban group. Comparing their abundances, the urbans are enriched in the three functions (*P* = 8.1*e*−15, *P* = 3.6*e*−15, and *P* = 1*e*−15, respectively, Wilcoxon rank-sum test, Figure 4D). The di-/oligosaccharides metabolism of US individuals is mainly driven by gene families associated with lactose utilization, which were considerably reduced in the microbiome of the traditional groups (*P* < 2.2*e*−16, Wilcoxon rank-sum test, Figure 4D). We also observed differences in metabolic pathways related to cofactors, vitamins, prosthetic groups, and pigments among the groups, the major gene families found in the microbiome of the Yanomami/Brazil and Yanomami/Venezuela were associated with folate/pterines, in the Matses and Tunapuco was riboflavin, and in the US group was tetrapyrroles (Supplementary Figure 3).

Interestingly, at level 1, the microbiome of the Yanomami/Brazil was distinct from the other traditional groups due to its significantly higher abundance (*P* < 0.05, Wilcoxon rank-sum test) of gene families involved in regulation/cell signaling, motility/chemotaxis, and virulence (Figure 4E). The regulation/cell signaling functions in the Yanomami/Brazil are driven by the abundance of programmed cell death and toxin–antitoxin systems, while the motility/chemotaxis function is driven by the presence of genes involved in flagellar motility in prokaryotes. The most abundant virulence function at subsystems 3 in the Yanomami/Brazil is cobalt, zinc, and cadmium resistance.

## Discussion

The gut microbiome is a diverse ecosystem with multiple metabolic and immune functions associated with the host diet and lifestyle (Gupta et al., 2017; Zinöcker and Lindseth, 2018). Therefore, considering the current variety of diets and lifestyles in human society, there are drivers of the gut microbiome yet to be accessed and explored to understand how they affect this ecosystem. The study of human groups that still maintain traditional modes of subsistence (hunter–gatherers and rural agriculturalists) provides valuable information regarding the microbiome before the urbanization and industrialization impact in human diet and lifestyle.

Therefore, in the present study, we characterized taxonomically and functionally the gut microbiome of 15 semi-isolated Yanomami individuals from Brazil, and compared with other South American traditional groups (uncontacted Yanomami from Venezuela, the Matses, and the Tunapuco) as well as an urban-industrialized group (United States) (Human Microbiome Project Consortium, 2012; Clemente et al., 2015; Obregon-Tito et al., 2015; Lloyd-Price et al., 2017). In this way, our study enlarged the number of Yanomami individuals analyzed, as well as hunter–gatherer groups, and contributed to a better comprehension of aspects concerning their microbiome functionality. The traditional groups studied here explore different niches of the Amazon Region, and contrast with the US group, which lives in a densely populated urbanized and industrialized society with access to medical care and high hygiene standards.

Consistent with previous studies (De Filippo et al., 2010, 2017; Yatsunenko et al., 2012; Schnorr et al., 2014; Clemente et al., 2015; Martínez et al., 2015; Obregon-Tito et al., 2015; Rampelli et al., 2015; Gomez et al., 2016; Mancabelli et al., 2017; Li et al., 2018; Rothschild et al., 2018), our analysis point to a higher bacterial diversity in the traditional groups, with taxonomic and functional features that distinguish them from urban-industrialized individuals. Microbiomes with high diversity have been showed to have a positive association with health, as consequence of the presence of a higher global metabolic potential, providing the host with a wide range of health-relevant metabolites (Heinken and Thiele, 2015; Larsen and Claassen, 2018).

The overall taxonomic patterns observed in the gut microbiome of the traditional South American groups resemble the gut microbiome profiles of traditional groups from Western, Central and Eastern Africa, as the children from Burkina Faso (De Filippo et al., 2010, 2017), the Hadza from Tanzania (Schnorr et al., 2014), and the BaAka pygmies from Central African Republic (Gomez et al., 2016). These groups are also more diverse than the urbans, are enriched in Proteobacteria, and present Spirochaetes (*Treponema* and *Brachyspira*) that are depleted in industrialized populations (De Filippo et al., 2010, 2017; Yatsunenko et al., 2012; Schnorr et al., 2014; Martínez et al., 2015; Rampelli et al., 2015; Gomez et al., 2016; Mancabelli et al., 2017). These similarities occur despite the South Americans and the Africans belong to distinct ethnic groups, and live and explore quite different ecosystems, but they maintain a traditional mode of subsistence and do not have access to processed and refined food in their daily diet (Gupta et al., 2017). This indicates that population lifestyle is a major determinant of the gut microbiome composition and diversity, overruling genetic backgrounds and geographic origin. Notwithstanding the lifestyle associated taxonomic profile of the South American traditional groups, each group presented a specific set of biomarkers. Interestingly, some biomarkers converge in their functional profile, e.g., *Roseburia*, *Anaerostipes*, *Eubacterium*, *Flavonifactor*, biomarkers of the Yanomami/Brazil, Yanomami/Venezuela, Matses, and Tunapuco, respectively, are butyrate-producing bacteria. Butyrate is an anti-inflammatory short chain fatty acid (SCFA) that induces mucin synthesis, contributing to colon health and gut integrity (Burger-van Paassen et al., 2009; Rivière et al., 2016). In contrast, the urban-industrialized biomarkers produce SCFAs other than butyrate, such as propionate, acetate, and succinate, which, in high proportions, may increase gut permeability, leading to a further unhealthy status (Brown et al., 2011). The Yanomami/Brazil and Yanomami/Venezuela have also lactic acid bacteria as biomarkers: *Enterococcus* and *Streptococcus.* Lactic acid bacteria is the group most commonly used in probiotics and these bacteria supply many beneficial traits in the gut ecosystem (Pessione, 2012; Azad et al., 2018). Besides being a lactic acid bacteria, *Enterococcus* are also mucin degraders, as well as *Akkermansia*, another biomarker of the Yanomami/Brazil group, which has been associated with healthier metabolic status and better clinical outcomes (Png et al., 2010; Dao et al., 2016).

Broader levels of functional categories poorly discriminated traditional and urban-industrialized groups. Interestingly, the stratification of these categories clearly segregated those groups. At level 1, the most expressive difference observed between traditional and urban groups is the high abundance of gene families associated to membrane transport in the latter. High abundance of membrane transport functions has been shown in urban societies, as the Norman (Obregon-Tito et al., 2015), agricultural western-like communities, as the Bantu (Gomez et al., 2016), and has been associated with high fat diets (Hildebrandt et al., 2009). At level 3, differences were identified within monosaccharides metabolism, where the main functions in the traditionalists and urbans were xylose and mannose metabolism, respectively. One of the main functions of the human gut microbiome is to extract energy from complex proteins and complex carbohydrates the human host do not digest, as fibers and other plant-derived polysaccharides (Flint et al., 2012). Xylans and mannans, polysaccharides of xylose and mannose, are the two major classes of hemicelluloses that accumulate in plant secondary walls (Scheller and Ulvskov, 2010). Interestingly, recent studies with mouse models revealed that mannose increased the Bacteroidetes to Firmicutes ratio in the gut, a characteristic observed in urban-industrialized groups (Sharma et al., 2018). On the other hand, *Treponema*, a prevalent genus in traditional populations that consume polysaccharide-rich diets, is a key xylan-degrader (Flint et al., 2008). Another difference observed in the United States versus traditional groups was the lactose utilization, which is enriched in the former and depleted in the latter group. This difference may be related to the lack of intake of dairy in the traditional groups (Obregon-Tito et al., 2015). Within the traditional groups, there were differences at level 3 of biosynthesis of vitamins: both Yanomami groups presented an enrichment in folate biosynthesis while the Matses and Tunapuco presented an enrichment in riboflavin biosynthesis, as well as in the US group. Riboflavin is the most commonly synthesized vitamin in the gut (Magnúsdóttir et al., 2015) and has been associated with the immune response through the activation of T cells (Kjer-Nielsen et al., 2012). Folate is associated with high-fiber and low-fat diets (Chan et al., 2019), which agrees with Yanomami diet from the present study.

Interestingly, the microbiome of the Yanomami/Brazil is unique concerning the presence of higher levels of functions associated with virulence, driven by the cobalt, zinc, and cadmium resistance. Cobalt is commonly distributed in nature and has a biological role as a metal constituent of the vitamin B12; however, excessive exposure induces adverse health effects (Leyssens et al., 2017). Zinc is an essential nutrient and plays a role in gene expression, biomolecular activity, and structural DNA stabilization (Bruins et al., 2000). Cadmium is a non-essential element, representing an environmental hazard to human health when contaminates the food chain, causing cumulative toxic effects in diverse human organs (Hyder et al., 2013). Cadmium and zinc are present in mine discharges, which disperses into the air, water, and soils, contaminating areas nearby mines (Xue et al., 2017). However, in Yanomami/Brazil area, cadmium contamination may occur as a consequence of the continuous discharge of batteries anywhere by the Yanomami along decades. These findings corroborate that the lifestyle as well as the ecosystem are some of the driving forces in shaping the gut microbiome (Schnorr et al., 2014; Gomez et al., 2016; Gupta et al., 2017).

## Conclusion

Exploring the gut microbiome of traditional groups is challenging, mainly due to the difficulty to access them. However, the study of these groups is essential, since they are living representatives of ancestral behaviors/dietary lost for a long time in the urban-industrialized groups. Here, it was revealed that even within very close and related traditional groups (as Yanomami/Brazil and Yanomami/Venezuela), there are biomarkers, and taxonomic and functional differences that distinguish and characterize their gut microbiome. This diversity may be a reflection of their nomadic behavior and the niches explored in the vast rainforest, despite their shared cultural and genetic background. Overall, the hunter–gatherers from South America and Africa present lifestyle associated microbiome, even though each of them harbors unique features. In order to better understand the microbiome aspects associated with health and disease, temporal and spatial studies considering larger number of samples from human groups with distinct lifestyles should be performed.

## Data Availability

The datasets generated for this study can be found in the NCBI under the BioProject PRJNA527208.

## Ethics Statement

The protocol of this study was reviewed and approved by the Oswaldo Cruz Foundation’s Ethics Research Committee No. 638/11 and by the National Ethics the Committee in Research – CONEP No. 16907.

## Author Contributions

JO-F collected the samples. LC processed the samples and analyzed the data. LC and AV interpreted the data and drafted the manuscript. All authors revised the manuscript, approved the final version to be published, and agreed to be accountable for the work.

## Conflict of Interest Statement

The authors declare that the research was conducted in the absence of any commercial or financial relationships that could be construed as a potential conflict of interest.
