# Supplementary figures and images for: Gut Microbiome Biomarkers and Functional Diversity Within an Amazonian Semi-Nomadic Hunter–Gatherer Group
Source: Front Microbiol. 2019 Jul 30;10:1743. doi: 10.3389/fmicb.2019.01743 (PMC6682603; doi:10.3389/fmicb.2019.01743)

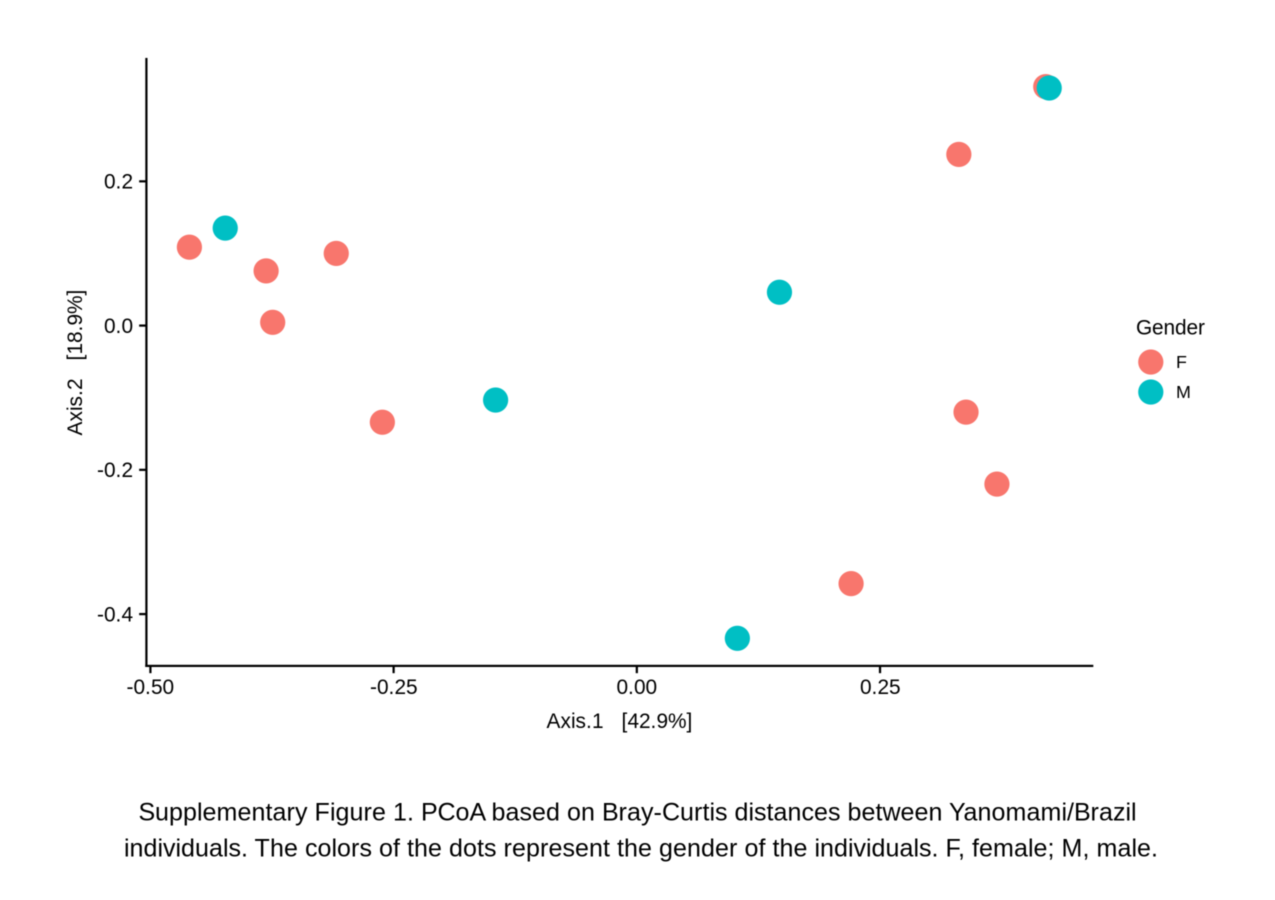

Supplement: Supplementary file 1 [file Image_1.TIFF]

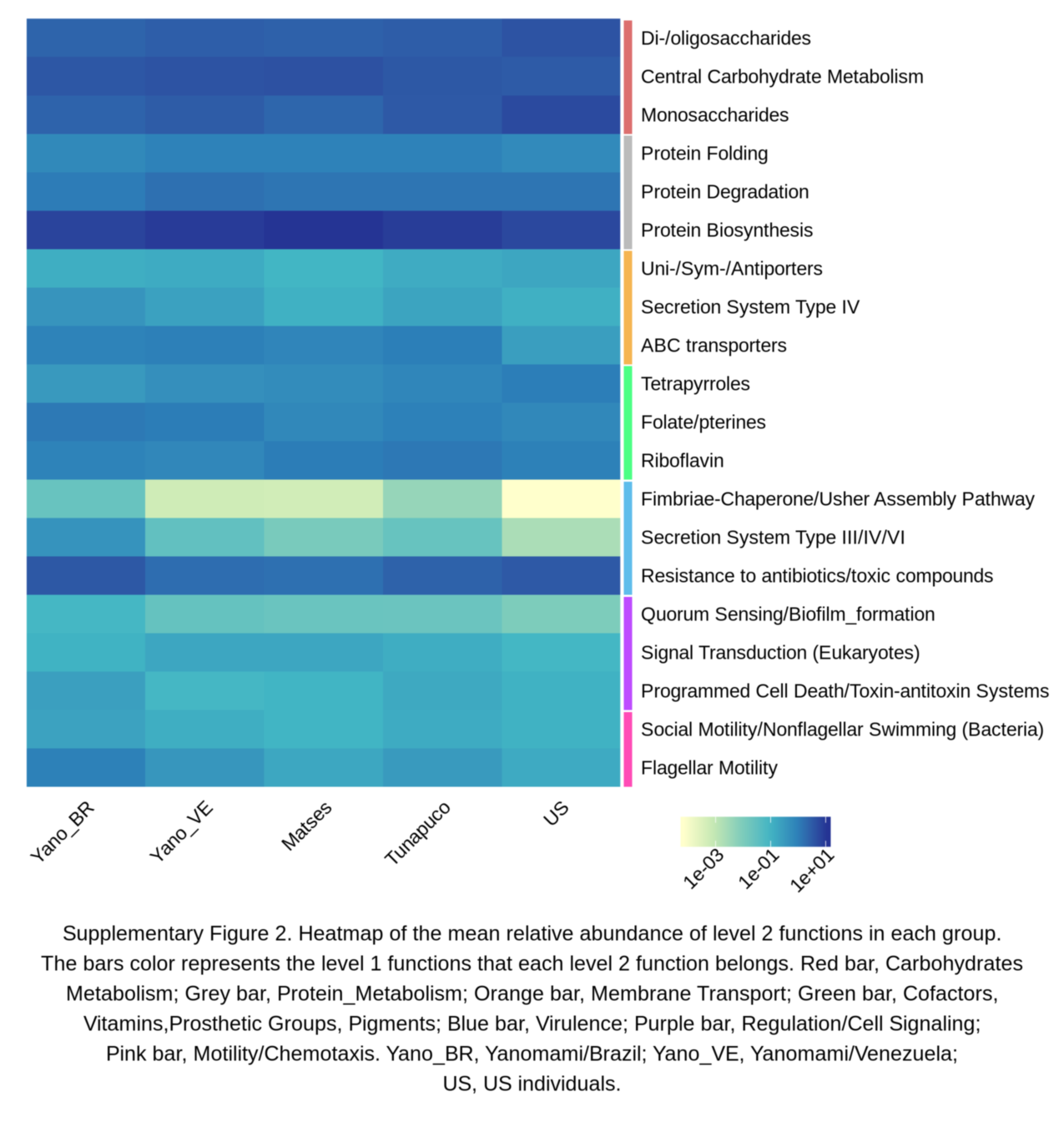

Supplement: Supplementary file 2 [file Image_2.TIFF]

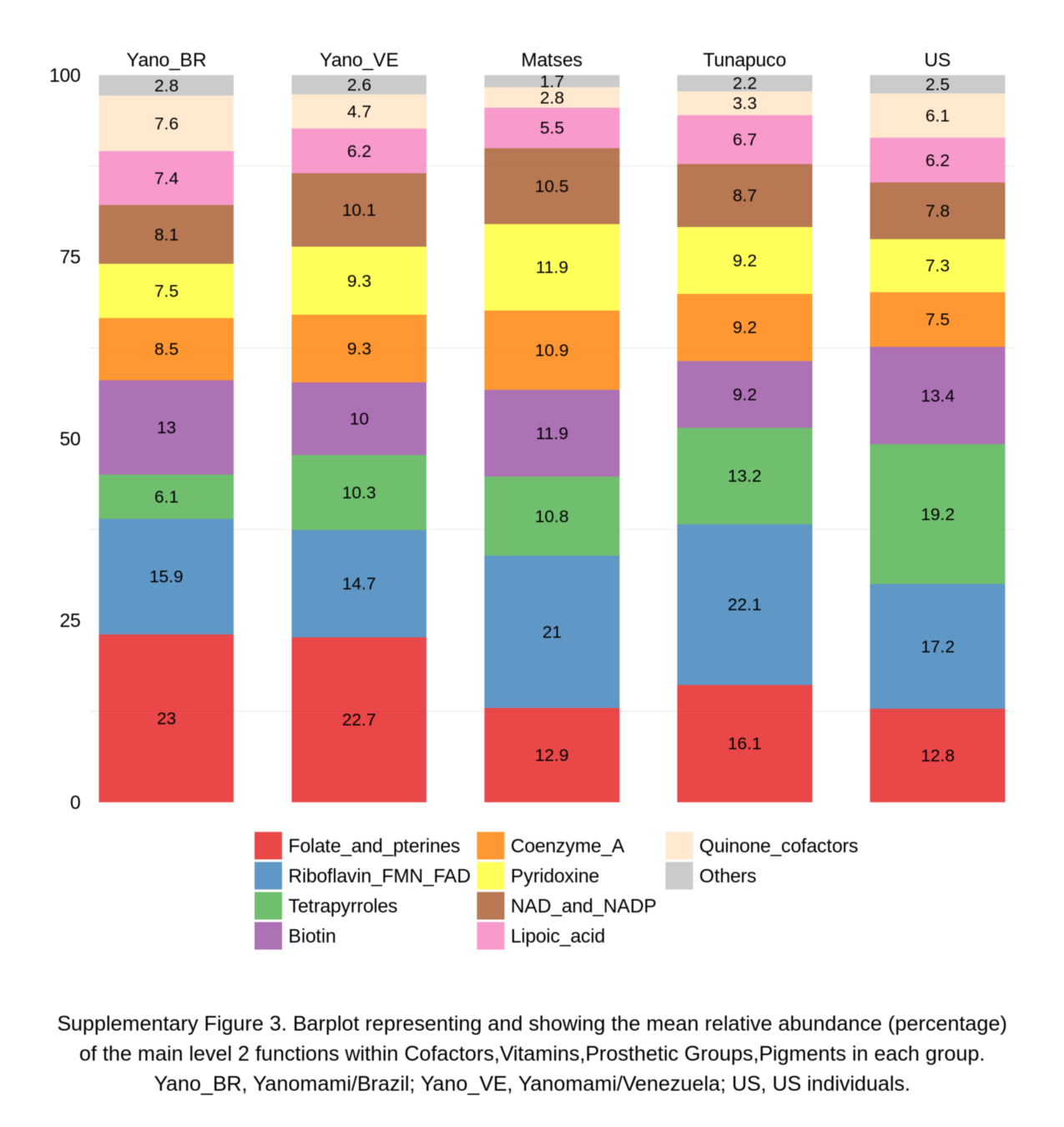

Supplement: Supplementary file 3 [file Image_3.TIFF]
